# Traditional vs Innovative Sanitizers: *In Vitro* and *In Situ* Challenge in Multidrug-Resistant Bacteria

**DOI:** 10.1021/acsomega.5c08705

**Published:** 2026-03-18

**Authors:** Gabriella Rayane Aparecida Ferreira, Letícia Roberta Martins Costa, Jéssica Laura Miranda, Micaela Guidotti Takeuchi, Mariana Comassio Chueiri, Carolyne Ferreira Dumont, Tanaje Luiz Izidio Ferreira de Amorim Junior, Rosineide Marques Ribas, Daise Aparecida Rossi, Gabriela de Paiva Loures, Hebreia Oliveira Almeida Souza, Mario Machado Martins, Luciana Machado de Bastos, Maria Abadia da Silva Celestino, Roberta Torres de Melo

**Affiliations:** † Laboratório de Biotecnologia Animal Aplicada, Faculdade de Medicina Veterinária, 28119 Universidade Federal de Uberlândia, Uberlândia 38405-302, Brazil; ‡ Lima & Pergher Ind. & Com., Av. Aírton Borges da Silva, 740 Distrito Industrial, Uberlândia, MG 38402-100, Brazil; § Laboratório de Microbiologia Molecular, Instituto de Ciências Biomédicas, Universidade Federal de Uberlândia, Uberlândia 38405-320, Brazil; ∥ Laboratório de Nanobiotecnologia Prof. Dr. Luiz Ricardo Goulart Filho, Instituto de Biotecnologia, Universidade Federal de Uberlândia, Uberlândia 38402-022, Brazil

## Abstract

Multidrug-resistant (MDR) bacteria pose a threat to public health worldwide. In this context, disinfectants are used to prevent and control the spread of these pathogens in hospital and industrial environments. The objective of this study was to determine minimum bactericidal concentrations (MBC) for 10 traditional and innovative active ingredients against 11 bacterial species. At the same time, the efficiency test was applied *in vitro* and the effect on hospital surfaces was analyzed *in situ*. The most promising compounds were selected to determine their mechanism of action in the Gram-positive bacterium (*Staphylococcus aureus*—MRSA) and the Gram-negative bacterium (*Pseudomonas aeruginosa*—PA) by metabolomic analysis. Resistance to 50% of the active ingredients was observed in the five strains, and PA was resistant to 70% of them. Peracetic acid, chlorhexidine digluconate, and neem extract eliminated all strains within 1 min in MBC. In efficiency tests, traditional products killed bacteria, with an average reduction of 7.07 ± 0.331 log cycles. Neem extract achieved an average reduction of 6.60 ± 0.33 log across all strains. *In situ*, peracetic acid did not allow bacterial growth, while biguanide, neem extract, tea tree oil, and orange oil achieved a reduction of more than 4 cycles of bacterial control on the surface. The exposure of MRSA and PA to the most promising compounds promoted distinct alterations in intra- and extracellular metabolism, which were modulated by the bacterial cell wall structure. The study highlights the importance of evaluating the practical efficacy of disinfectants, emphasizing the diversity of approaches including natural compounds as alternatives in critical environments.

## Graphical abstract

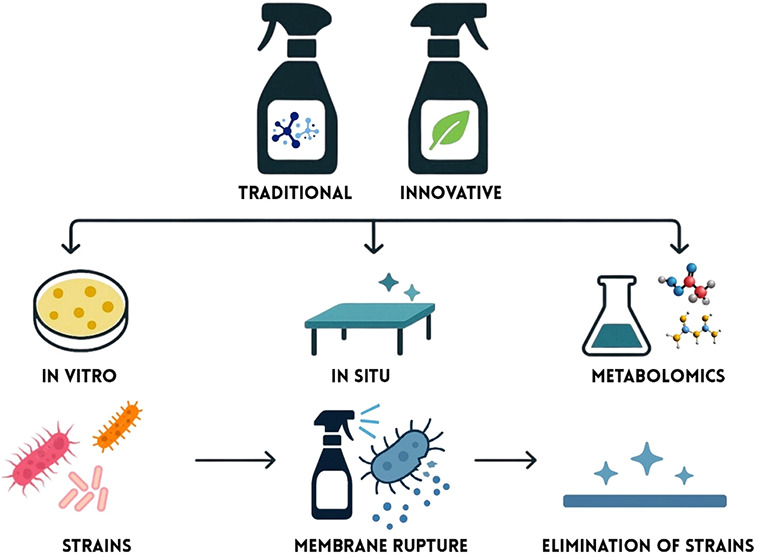

## 1 Introduction

The steady increase in the emergence and spread of multidrug-resistant (MDR) bacteria in hospital settings represents a serious concern, persisting as a significant challenge for infection control and hospital epidemiological practice on a global scale.^1^ Concomitantly, in the scenario of the food industries, there has been an increase in concern about microbial contamination and the risks of infections associated with the consumption of contaminated food, especially that of animal origin.^2^ Bacterial species including *Klebsiella pneumoniae*, *Pseudomonas aeruginosa*, *Acinetobacter baumanii*, and methicillin-resistant *Staphylococcus aureus* (MRSA), play a relevant role in morbidity and mortality in cases of nosocomial infection due mainly to multidrug resistance.^3^

In parallel, the main etiological agents involved in foodborne diseases of animal origin on a global scale include the genera *Salmonella* spp., *Campylobacter* spp., and the species *Escherichia coli*.^4^ *Salmonella* and *E. coli* accounted for 10.9 and 32.3% of the cases registered in Brazil, respectively, from 2013 to 2022,^5^ and *Campylobacter* under-reported in the country,^6^ but the main food zoonosis in Europe with 137,107 cases.^7^

Effective control of MDR strains is essential to preserving the efficacy of antimicrobial treatments in hospitals and reducing the risks of contamination in industries. The proper cleaning and disinfection procedure of hospital and industrial environments and surfaces can be associated with the application of biocidal agents,^8^ of which the most widely used include quaternary ammonium, chlorine, peracetic acid, and biguanide.^9^

Natural products, such as essential oils, have been used in the control of microorganisms due to their antiseptic properties, including biocide, fungicide, and virucide. These characteristics have been explored over many years, and currently, the use of these oils has gained prominence.^10^ Its application in industry and hospital environments emerges as an effective preventive or control measure to prevent or inhibit the growth of pathogens.^11^

Our approach included the analysis of the *in vitro* antimicrobial effect of 10 sanitizing agents, of which 5 are traditionally known and 5 are innovative from biodegradable raw materials. The study included the verification of the effect of the compounds on 11 bacterial strains, of which 8 are MDR of hospital and industrial importance. The investigation extended to an *in situ* study on contaminated surfaces of the hospital environment to determine the most effective agents for microbial control and for the analysis of the mechanisms of action of the most promising compounds.

## 2 Material and Methods

The private sector company undertook a study together with our team with the purpose of analyzing the raw materials used in the manufacture of products intended for the elimination of bacteria. In this context, tests were conducted involving a total of 10 different raw materials, divided equally into 2 sets: 5 of them representing types already consolidated and marketed, while the other 5 referred to innovative agents, not yet launched but planned for future introduction on the market.

### 2.1 Strains and Conditions

The study was conducted with 11 bacterial strains, of which three are standard ATCC strains and 8 are multidrug-resistant strains maintained in the strain bank of the Molecular Epidemiology Laboratory of the Federal University of Uberlândia (LEPMOL). The strains were sourced from the environment of the Veterinary Hospital of the Federal University of Uberlândia (VH), the Clinical Hospital of the Federal University of Uberlândia (CH) and partner poultry exporting industries (PI), as described in Table 1.

**Table 1:** Description of the Strains Used in the Study Including Origin, Year, Resistance Class, and Form of Identification^a^

Id	species	origin	year	resistance classes
EC^b^	*E. coli* ATCC 25922			
SA^b^	*S. aureus* ATCC 25923			
SE^b^	*Salmonella enteritidis* ATCC 13076			
AB	*Acinetobacter baumannii*	VH/PI	2021	aminoglycoside, β-lactam, fluoroquinolone, and carbapenem
KPC	*K. pneumoniae* carbapenemase	CH	2012	fluoroquinolone, cephalosporin, β-lactam, and carbapenem
MRSA	methicillin-resistant *S. aureus*	CH	2011	β-lactam, aminoglycoside, fluoroquinolone, macrolide
PA	*P. aeruginosa*	CH	2014	β-lactam, aminoglycoside, fluoroquinolone, and carbapenem
CJ	*Campylobacter jejuni*	PI	2015	macrolide, fluoroquinolone, tetracycline, aminoglycoside, β-lactam
ECA	*E. coli* APEC	PI	2019	cephalosporin, β-lactam, sulfonamide, and tetracycline
SC	*Salmonella choleraesius*	PI	2017	fluoroquinolone, β-lactam, sulfonamide, and tetracycline
ST	*Salmonella typhimurium*	PI	2013	fluoroquinolone, β-lactam, tetracycline, and sulfonamide

a Id: identification.

b Control strains.

The strains were reactivated from Nutrient Agar in BHI broth (Kasvi) and maintained at 36 °C for 24 h, followed by cultivation on Soybean Tryptone Agar (TSA) (Oxoid)/Standard Plate Count Agar (PCA) (Oxoid) for 24 h at 36 °C. For *Campylobacter*, reactivation was performed on Campylobacter Blood-Free Selective Medium CCDA Agar (Neogen) and incubated in microaerophilia (Probac) at 37 ± 1 °C for 48 h.^12^

### 2.2 Chemicals

The products used in this study were acquired and selected during the period from July 2021 to June 2023 through a partnership with a company that produces chemical sanitizing agents located in the city of Uberlândia-MG, which determined the test concentrations to be applied. Ten chemical compounds were used, of which five represent active ingredients traditionally used in the routine cleaning of hospital and industrial environments and five were recently introduced and not yet used in the products supplied by the company, which have active ingredients with natural and biodegradable characteristics (Table 2).

**Table 2:** Sanitizers Used and Their Respective Concentrations Preliminarily Tested^a^

agents type	Id	agents name	tested concentrations (%)
traditional	T1	peracetic acid 15%	0.1; 0.2; 0.3; 0.4; 0.5; 0.6; 0.7; 0.8; 0.9; and 1
	T2	Quatercap BDD 500 g/BDD 800 (quaternary ammonia) 80%^b^	
	T3	Quatercap DBB 800/ARQMC 210 (quaternary ammonia) 80%^b^	
	T4	Vantocil (biguanide) 20%	
	T5	digluconate chlorhexidine 20%	0.15; 0.3; 0.45; 0.6; 0.75; 0.9; 1.05; 1.2; 1.35; and 1.5
innovative	I1	Olyo’s pines	0.7812, 1.5625, 3.125, 6.25, 12.5, 25, 50, and 100
	I2	neem extract	
	I3	tea tree oil	
	I4	orange oil	0.1; 0.2; 0.3; 0.4; 0.5; 0.6; 0.7; 0.8; 0.9; 1; and 100
	I5	lactic acid	

a Id: Identification.

b The molecules BDD (80% alkyl dimethyl benzyl ammonium chloride + 80% didecyl dimethylammonium chloride) and DBB (80% alkyl amimethyl methyl dimethylammonium chloride + 80% didecyl dimethylammonium chloride) molecules, are fifth generation biocides because they are a mixture involving first and fourth generation molecules, one product containing a higher percentage of the first generation quaternary and another of the fourth generation, both with distinct percentages.

### 2.3 Minimum Bactericidal Concentration (MBC)

MBC was determined using the microdilution technique according to the protocol of the Clinical and Laboratory Standards Institute.^12^ Briefly, a standardized bacterial suspension was prepared for each of the 11 strains tested at a concentration corresponding to 0.5 on the MacFarland scale, and 10 concentrations of the chemical agents were used (Table 3), with exposure periods ranging from 1 to 10 min. Every minute, an aliquot of 10 μL of each inoculum diluted in TSA or CCDA plates (in the case of *Campylobacter*) was added to verify MBC. The plates were incubated at 36 °C for 16–20 h.^12^ For *Campylobacter*, incubation was at 37 ± 1 °C for 48 h. All tests were performed in triplicate with negative controls composed of the medium without the addition of bacteria.

**Table 3:** MBC, Minimum Exposure Times and Resistance Parameters of the Strains in Relation to Chemical Agents Tested

		traditional agents					innovative agents						
strain		T1	T2	T3	T4	T5	I1	I2	I3	I4	I5	*R* *n*/*N* (%)	
EC^a^	MBC	—	—	0.3%	—	—	100%	25%	—	1%	0.3%		5/10
	*T*_exp_			1 min			1 min	1 min		1 min	2 min		(50)
SA^a^	MBC	—	—	—	—	—	100%	25%	—	NI	0.3%		4/10
	*T*_exp_						1 min	1 min			2 min		(40)
SE^a^	MBC	—	—	—	—	—	100%	25%	—	1%	0.3%		4/10
	*T*_exp_							1 min	1 min	1 min	2 min		(40)
AB	MBC	—	—	—	—	—	100%	25%	—	NI	0.4%		4/10
	*T*_exp_						1 min	1 min			1 min		(40)
KPC	MBC	—	—	—	0.2%	—	100%	25%	—	1%	0.3%		5/10
	*T*_exp_				2 min		1 min	1 min		1 min	2 min		(50)
MRSA	MBC	—	—	—	0.2%	—	100%	25%	—	NI	0.3%		5/10
	*T*_exp_				1 min		1 min	1 min			2 min		(50)
PA	MBC	—	0.9%	0.7%	0.3%	—	100%	25%	—	NI	0.3%		7/10
	*T*_exp_		10 min	10 min	4 min		1 min	1 min			2 min		(70)
CJ	MBC	—	—	—	—	—	100%	25%	—	1%	0.3%		4/10
	*T*_exp_						1 min	1 min		1 min	2 min		(40)
ECA	MBC	—	—	0.1%	—	—	100%	25%	—	1%	0.4%		5/10
	*T*_exp_			6 min			1 min	1 min		1 min	1 min		(50)
SC	MBC	—	—	—	—	—	100%	25%	—	NI	0.3%		4/10
	*T*_exp_						1 min	1 min			2 min		(40)
ST	MBC	—	0.2%	—	—	—	100%	25%	—	NI	0.4%		5/10
	*T*_exp_		1 min				1 min	1 min			1 min		(50)
Conc. set (%)		0.1	1	0.8	0.4	0.15	100	25	0.7812	1	0.4		
CRA (%)		0	2 (18,2)	3 (27,3)	3 (27,3)	0	11 (100)	11 (100)	0	11 (100)	11 (100)		

a *T*_exp_: Minimum exposure time, (—): indicates susceptibility at the lowest concentration and time evaluated; NI: did not inhibit in any of the concentrations and times used. Conc. set: concentration used in efficiency and *in situ* tests. *R*: resistance to at least one of the concentrations tested. *n*/*N*: number of active ingredients that the bacterium showed resistance/total. (%): Percentage of resistance to agents. CRA (%): number and percentage of strains resistant to the active.

To complement the analysis presented in Table 3 and provide a comprehensive view of the antimicrobial activity, we prepared Supporting Table S1, which compiles the MBC values obtained across all exposure times (1–10 min) for all bacterial strains evaluated. This table includes all concentrations tested for both traditional (T1–T5) and innovative (I1–I5) agents, allowing the temporal progression of bactericidal activity to be fully assessed and enabling direct comparison among the different compounds. The inclusion of these additional data provides a more robust interpretation of the inhibition profile, highlighting significant differences in the time required for each agent to achieve complete bactericidal activity.

### 2.4 Efficiency Testing

The modified tube macrodilution method was used.^13^ Briefly, the inoculum of 6–8 log CFU of each of the 11 test microorganisms was placed in direct contact with the 10 chemical agents at the minimum concentrations and times predefined in the MBC, prioritizing the shortest exposure time (1 min) due to practical applicability. The assays were performed in triplicate and after exposure, serial dilution, duplicate plating in PCA or CCDA (*Campylobacter*) and incubation at 36 °C for 24 h or 37 ± 1 °C for 48 h, respectively. The tests were considered satisfactory when there was a reduction of at least four log cycles of the initial count of the microorganisms evaluated using the mean values of the replicates.

### 2.5 *In Situ* Tests

Samples were collected daily or weekly for 5 months from procedure tables in the Intensive Care Unit of the Veterinary Hospital at the Federal University of Uberlândia. Using sterile swabs, samples were taken from three different regions, each covering 100 cm^2^, and stored in Letheen Broth (Oxoid) for transport to LEPMOL. The cleaning followed standard hospital procedures: washing with water and neutral detergent, followed by rinsing, drying, and application of disinfectant for 1 min. Swabs were then collected for analysis. Each sample had eight replicates, diluted, plated on PCA agar, incubated at 37 °C for 48 h, and bacterial counts compared to pre- and postdisinfection. Disinfectants were deemed effective if bacterial reduction was ≥99.99%.^14^

### 2.6 Metabolomic Analysis

Metabolomic analysis was performed on pure cultures of MRSA and PA, which were previously grown in BHI broth at three different time points (biological replicates) under the appropriate conditions. After growth, cells at the onset of the stationary phase were treated for 1 min with the most effective sanitizing agents (T1, I2, and I3). The samples were then collected by centrifugation at 8,000*g* for 10 min at 4 °C, washed with sterile saline solution, and subjected to metabolite extraction of both the pellet and the resulting supernatant. For the extraction of metabolites, 1000 μL of spectroscopic-grade methanol was added to the material and homogenized in a vortex for 5 min. The material was centrifuged for 15 min at 13,000*g*, and the supernatant was transferred to another Eppendorf, which was subjected to a vacuum concentrator for 30 min. The material was resuspended in 400 μL of methanol and filtered through a 0.22 μm filter.

Metabolite analyses were performed using an Agilent 7890B GC System/5977B GC/MSD technologies instrument. Separation was provided by a capillary column (Agilent DB-5HT 30 m × 250 μm × 0.25 μm) with high-purity helium at a constant flow rate of 1.2 mL min^–1^. The following gas chromatography (GC) conditions were used: the initial column temperature was maintained at 60 °C for 2 min, then increased to 280 °C at 5 °C min^–1^ and then maintained for 30 min. The mass spectrometer was operated in full scan mode from 50 *m*/*z* to 550 *m*/*z*. The transfer line to the mass spectrometer was heated to 240 °C and the quadrupole to 150 °C. The was 6 μL injection volume.

MassHunter Qualitative v 10.0 software was used to process raw data. A “Molecular feature extraction (MFE)” tool was used for extraction of the mass spectra and conversion to .CEF extension. Agilent Mass Profiler Professional (MPP) software v. B.13.1.1 was used to filter and analyze the extracted molecule compounds. The filters used were minimum absolute abundance = 5000 counts and all allowable charges. The analysis parameters were retention time tolerance of 0.15 min; mass window 15 ppm +2 mDa. Molecular compounds considered were present in 100% of at least one group. Metabolites lacking a validated bacterial pathway in KEGG and MetaCyc were excluded from the analysis.

### 2.7 Statistical Analysis

The data collected were submitted to descriptive statistical analysis, which involved the calculation of the percentages of agents that presented satisfactory results in each of the microbiological tests performed. The results obtained in efficiency and *in situ* tests were evaluated for Gaussian distribution by Kolmogorov–Smirnov or D’Agostino and Pearson tests. Then, the analyses that comprised two variables were compared using the unpaired *t* test. Three or more variables were submitted to one-way analysis of variance (ANOVA) analysis. All analyses were conducted using Graph Pad Prism 8.0.1 software, with a 95% confidence interval.

## 3 Results

### 3.1 Resistance Profiles and Definition of Minimum Bactericidal Concentration

It was identified that the percentage of active ingredient resistant strains (% CRA) was higher for natural products, so that four of the five (80%) products tested allowed the growth of all strains in at least one of the concentrations tested, except for tea tree oil (I3). On the other hand, this characteristic was identified only in three strains (27.3%) for T3 and T4 products and in two strains (18.2%) for T2.

Of the 11 strains evaluated, 5 were resistant to 5/10 (50%) of the active ingredients, 5 were resistant to 4/10 (40%) and PA was resistant to 7/10 (70%). The products, peracetic acid (T1) and chlorhexidine digluconate (T5) were active in all strains tested, since they were able to inhibit them at the lowest concentration tested and at the shortest exposure time (1 min). The MBC values were equivalent to 0.1 and 0.15%, respectively. The same profile was identified for tea tree oil (I3), so that exposure to 0.7812% for 1 min was sufficient to control the strains tested (Table 3).

As for the other traditional active ingredients, it was detected that Quatercap BDD 500 g/BDD 800 (T2) presented MBC values equal to the lowest concentration and time of action tested (0.1% per 1 min) for nine of the 11 strains evaluated. The exceptions were PA, whose MBC was 0.9% at 10 min, and MRSA, whose effect was identified at a concentration of 0.2% for 1 min. Quatercap DBB 800/ARQMC 210 (T3) was effective for bacteria AB, CJ, KPC, SC, SE*, ST, MRSA, and SA* at the concentration and time tested (0.1% for 1 min). However, for PA, ECA, and EC*, it was observed that the inhibition and the biocidal effect were only identified at concentrations of 0.7, 0.1, and 0.3% at 10, 6, and 1 min, respectively. Vantocil (T4) showed lower action in three bacterial strains, which included PA with MBC equivalent to 0.3% at 4 min, *S. aureus* with inhibition at 0.2% for 1 min, and KPC whose control was identified at a concentration of 0.2% at 1 min.

Neem extract (I2) exhibited activity in all tested strains at a concentration of 25%, inhibiting them at 1 min of exposure. Orange oil (I4) demonstrated 1% MBC after 1 min of contact with KPC, SE*, CJ, EC*, and ECA. There was no inhibition in any of the I4 concentrations and exposure times in the strains SA*, AB, MRSA, PA, SC, and ST. Lactic acid (I5) was effective in inhibiting the strains SA*, SE*, KPC, MRSA, PA, CJ, ECA, and SC a 0.3% in 2 min, with the exception of AB, ST, and EC*, which were inhibited to 0.4% in 1 min. As for Pinus oil (I1), antimicrobial activity was observed at a concentration of 100% for all strains.

In view of the results obtained, the specific concentrations for efficiency and *in situ* tests were defined, all performed with 1 min of exposure. For traditional T1 to T5 sanitizers, the concentrations were, respectively, 0.1% or 1000 ppm, 1% or 10,000 ppm, 0.8% or 8000 ppm, 0.4% or 4000 ppm, and 0.15% or 1500 ppm. For the I1 to I5 innovators, respectively, we had 100% or 1,000,000 ppm, 25% or 250,000 ppm, 0.7812% or 7812 ppm, 1% or 10,000 ppm, and 0.4% or 4000 ppm.

### 3.2 Efficiency of Traditional and Innovative Agents

There was no statistical difference in the bacterial inoculum used before exposure to the products (one-way ANOVA, *p* = 0.0773), so that a mean of 7.07 ± 0.31 log CFU was obtained for the 11 strains tested with the traditional active ingredients. Similarly, there was also no difference in the initial quantity of bacteria before contact with the innovative active ingredients (one way ANOVA, *p* = 0.9986), whose mean was 6.60 ± 0.33 log CFU. The mean values and deviations related to the inoculum concentration for each strain tested are shown in Figure 1.

**Figure 1:**
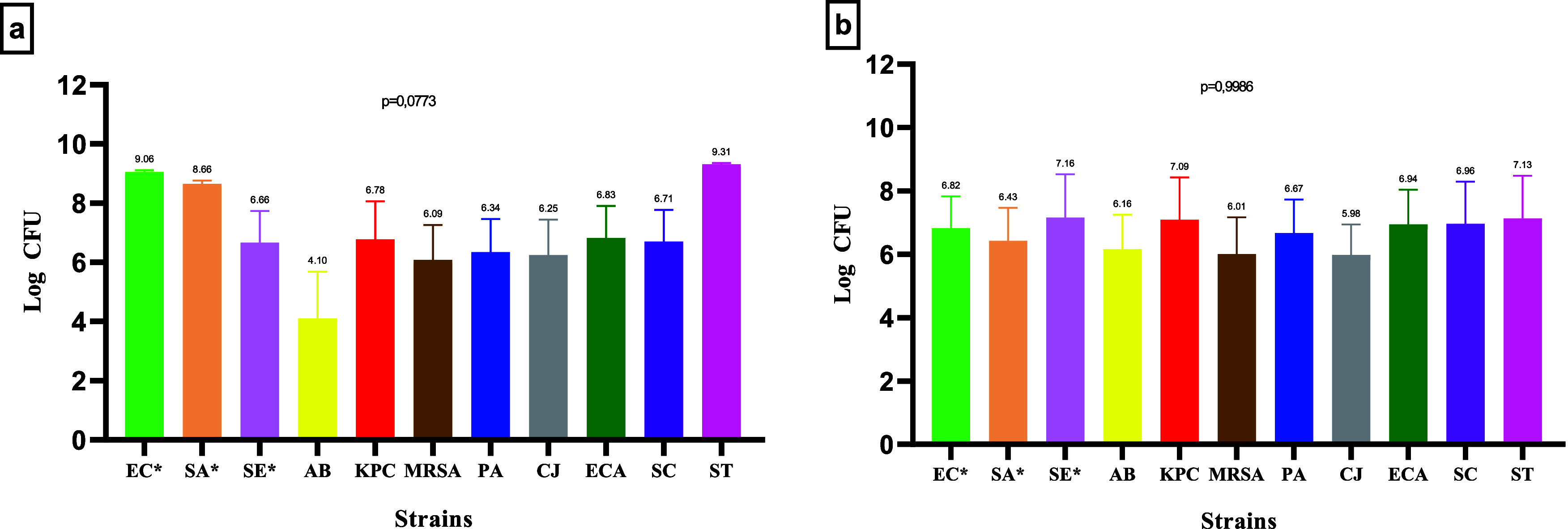
Bacterial count obtained in the initial inoculum for the efficiency test with traditional (a) and innovative (b) products. *p* > 0.05, one way ANOVA test.

After the test with the traditional products, we observed that there was efficiency in the removal of more than 4 log cycles (>99.99%) of the bacteria tested since there was no growth of any of the strains (*p* < 0.0001—Kruskal–Wallis). Consistent with a mean reduction of 7.07 ± 0.31 log CFU after contact with all products at the test use concentrations of T1 (0.1%), T2 (1%), T3 (0.8%), T4 (0.4%), and T5 (0.15%) in 1 min of exposure (Figure 2a).

**Figure 2:**
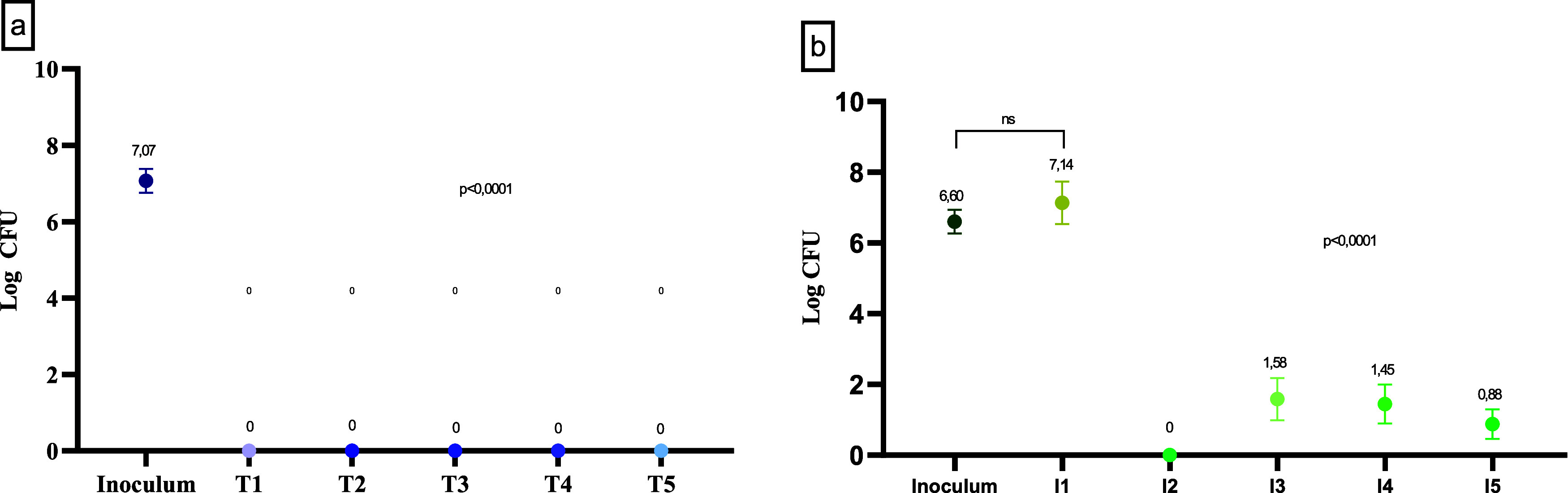
Bacterial count obtained after efficiency test with traditional (a) and innovative (b) active ingredients. *p* < 0.0001, significant difference in the one-way ANOVA test.

As for the innovative active ingredients, it is noted that neem extract (I2) was the most efficient in the disinfection process since no growth of any strain was obtained, equivalent to an average reduction of 6.60 ± 0.33 log CFU (>99.99%) (*p* < 0.0001—Kruskal–Wallis). Except for pine oil (I1), which showed no effect (*p* > 0.9999—Kruskal–Wallis), the other compounds were also able to remove more than 99.99% of the average value found for all bacteria tested, equivalent to a reduction of 5.02, 5.15, and 5.72 log cycles for I3, I4, and I5, respectively (Figure 2b).

By discriminating the data regarding the effect by bacteria for each innovative active ingredient, it was identified that pine oil (I1) did not show efficiency in any of the strains tested, since the bacterial count did not vary significantly (*p* = 0.5414) with a maximum reduction of 1.64 log CFU in SE* and a maximum bacterial multiplication of 2.67 log CFU in AB (Figure 3a).

**Figure 3:**
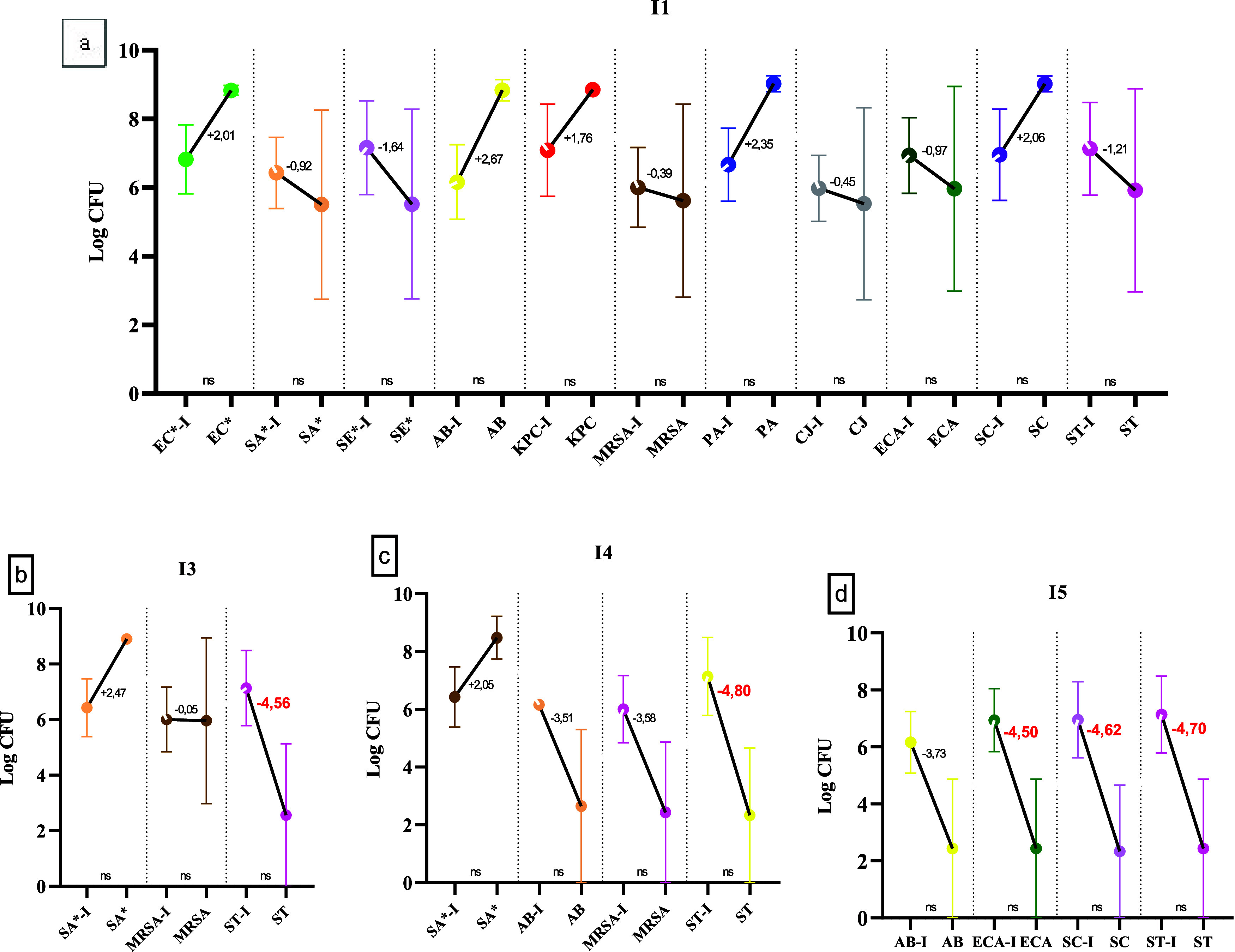
Means and standard deviations of the microbiological counts broken down for viable bacteria obtained in the efficiency test for natural products I1 (pine oil) (a), I3 (tea tree oil) (b), I4 (orange oil) (c), and I5 (lactic acid) (d). ±: bacterial growth/reduction in log CFU (red indicates efficiency >99.99%). ns: statistical equivalence in the Student’s *t* test (*p* > 0.05). Bacterial strains not included in the graphs indicate their inhibition in the efficiency test.

Susceptibility was detected in the absence of microbial growth of PA, KPC, and CJ in contact with I2, I3, I4, and I5. Despite the presence of ST after contact with the four active ingredients (I1, I3, I4, and I5), there was a reduction of more than 99.99% of this strain in I3, I4, and I5. The inefficiency of I3 (tea tree oil) and I4 (orange oil) for the control of MRSA and SA* suggests less effect against Gram-positive bacteria (Figure 3b,3c), beyond growth of AB in I4.

For I5, the feasibility of AB, ECA, SC and ST suggests some resistance factor in Gram-negative MR to antibiotics, but the product proved to be efficient for SC, ST, and ECA, with a reduction of 4.62; 4.70, and 4.50 log cycles, respectively (Figure 3d).

### 3.3 *In Situ* Testing of Traditional and Innovative Agents

The general analysis of the results showed that the counts obtained before the cleaning process did not fluctuate in any of the repetitions performed (*p* = 0.2299—one way ANOVA) and were equivalent to an average of 7.27 ± 0.20 log CFU. Except for active T3, the others showed a statistically significant difference in relation to the control (before) (Figure 4).

**Figure 4:**
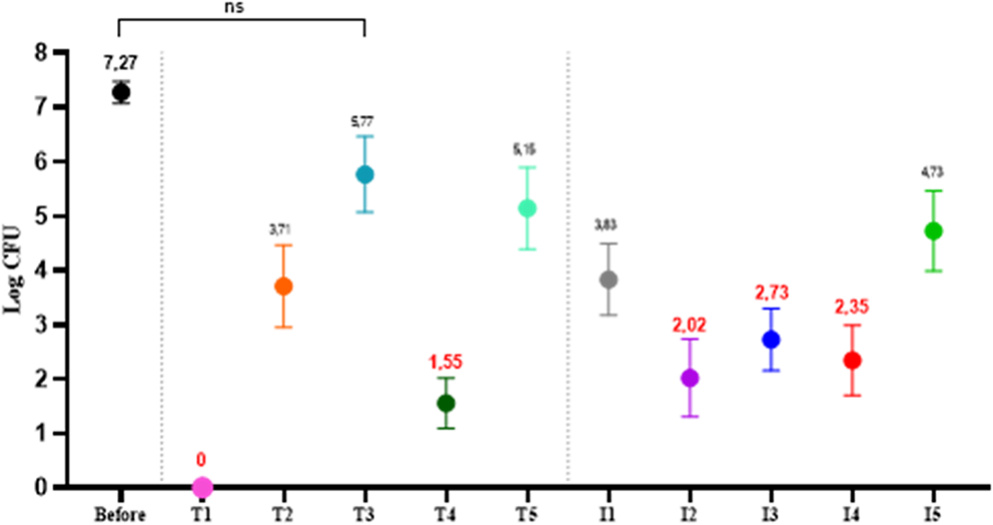
Means and standard deviations of the microbiological counts obtained in the *in situ* test at the VH-UFU. T: Traditional agents. N: innovative active ingredients. Averages marked in red indicate efficiency >99.99%. Statistical equivalence in the one-way ANOVA test (*p* > 0.05).

Of the five traditional agents, two were efficient (40%). Innovative agents, on the other hand, obtained 3/5 efficiency (60%). The T1 product was the best in the disinfection process since we did not obtain bacterial growth after its application (*p* < 0.0001—one way ANOVA). This makes evident the ability to remove more than 99.99% of bacteria, considering the reduction of more than 7 log CFU. The T4, I2, I3, and I4 products also showed efficiency >99.99%, since they showed a reduction of 5.72; 5.25; 4.54, and 4.92 log cycles, respectively (Figure 4).

### 3.4 Metabolic Effects

The analysis of the metabolites in all assays allowed us to identify a total of 906 signals expressed in both MRSA and PA in all treatments. After applying the 100% frequency of occurrence filter, this panel was reduced in a specific way for each bacterium and in each test, indicating only the signs detected in all samples. Comparisons between the treated group (exposed for 1 min to T1, I2 and I3) to the control group (MRSA or PA in suspension) performed using the unpaired *t* test, *p* < 0.05, combined with an absolute fold change ≥ 2, which allowed the selection of the number of metabolites differentially expressed in each condition. Finally, the mapping in KEGG and MetaCyc databases allowed to discriminate only compounds with bacterial metabolic pathways involved.

After 1 min of T1 exposure, MRSA maintained 57 intracellular metabolites detected in all replicates, 10 of which showed significant variation and 7 bound to bacterial metabolic pathways (Figures 5a and 6a). An increase in Cer­(d18:0/14:0), Ala-Leu-Ala, Hydroxyprolyl-Leucine, Stigmatellin Y, Hydroxypentobarbital, Tiapamil, and Arachidonoyl-EA­(d8), oxidative product signals and redirection of lipid synthesis to saturated chains, while C16-Sphinganine, *trans*,*trans*-Farnesol, and Ala-Pro-Val were reduced, showing oxidative cleavage of sphingolipids, quorum sensing suppression and selective extravasation of peptides. In the supernatant (Figures 5b and 6b), 51 metabolites were constant, 8 varied, and were present in an increased form, and included Ala-Leu-Ala, Thr-Gly-Pro, l-β-Asp-Leu, (+)-3-hydroxy-behenic, 8,8-diethoxy-2,6-dimethyl-2-octanol, Hydroxypentobarbital, and Eudesmin, confirming intense permeabilization of the bilayer.

**Figure 5:**
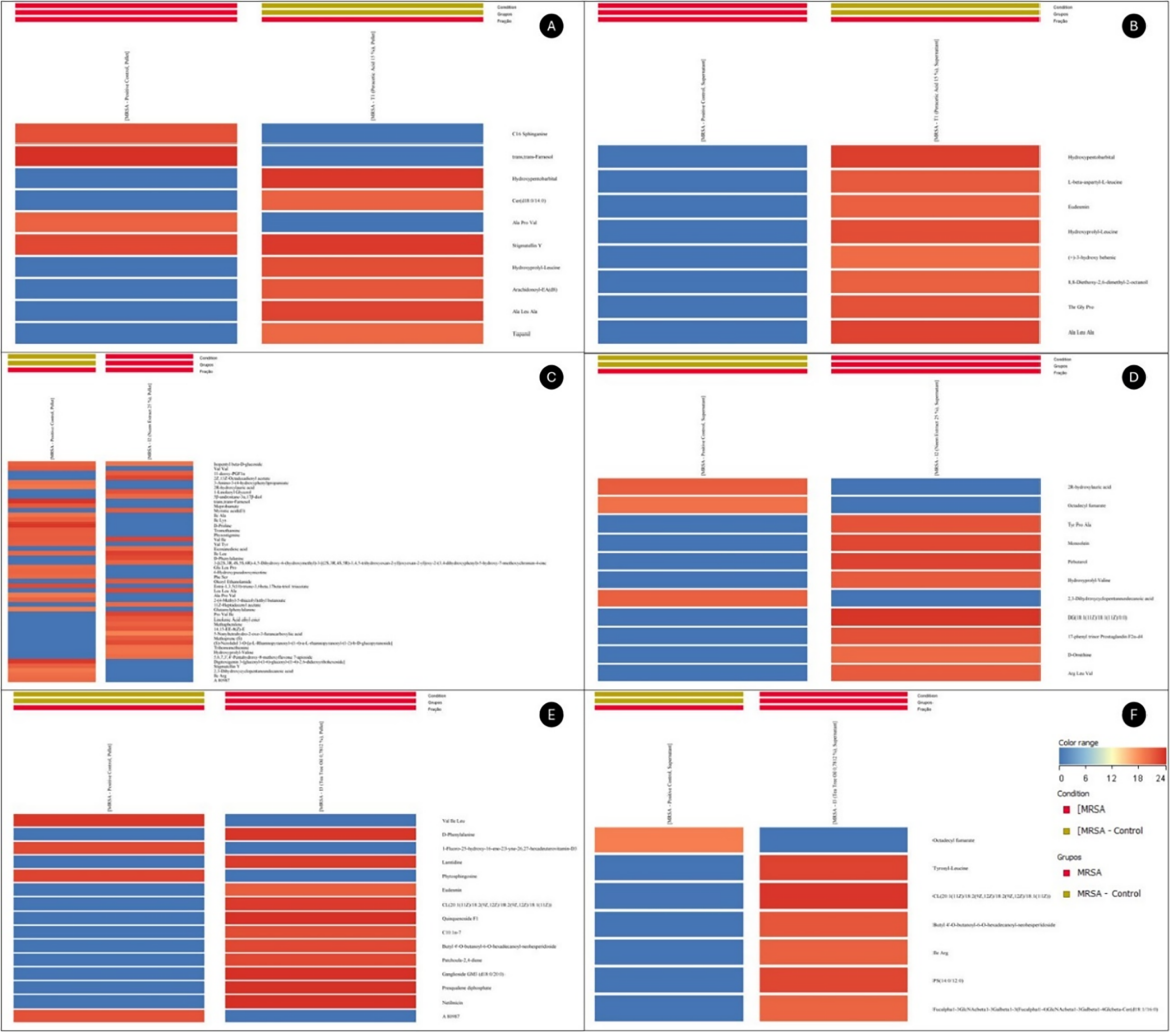
Heatmaps representing the relative abundance mean of selected metabolites in pellets and supernatants of MRSA in T1 (A, B), in I2 (C, D), and in I3 (E, F), respectively.

**Figure 6:**
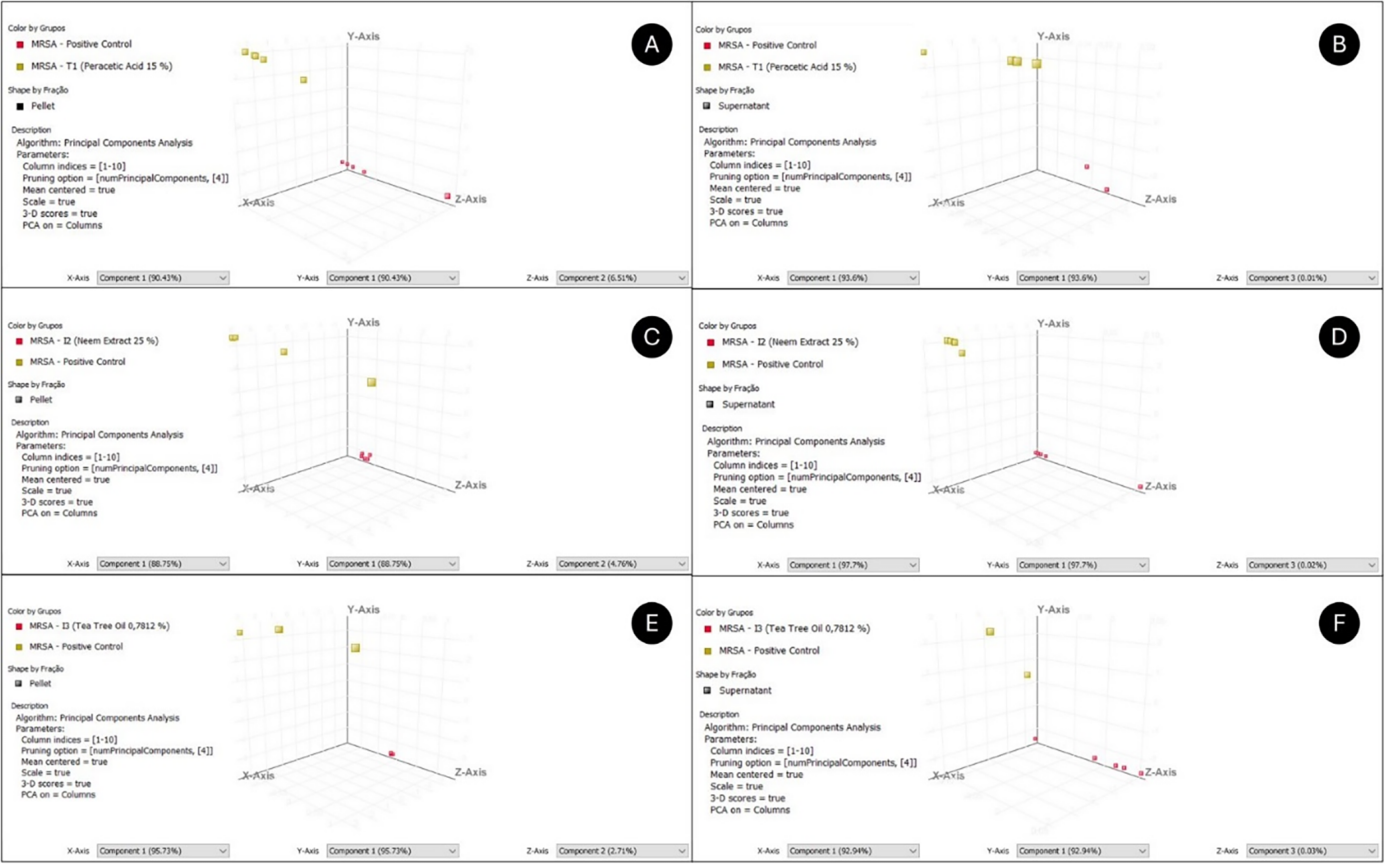
Principal Component Analysis (PCA) score plot based on metabolomic profiles of pellets and supernatants of MRSA in T1 (A, B), in I2 (C, D), and in I3 (E, F), respectively.

In parallel, PA produced 41 universal intracellular metabolites in the presence of T1 (Figures 7a and 8a), but only 2 were differential and bound to membrane lipid pathways and terpene signaling, which are nitropolyzonamine and (5α,8β,9β)-5,9-epoxy-3,6-megastigmadien-8-ol, respectively, whose production was reduced, suggesting redox modulation and inhibition of quorum sensing. In the PA supernatant (Figures 7b and 8b), 36 metabolites remained in all samples and 6 varied, with an elevation of Leu-Phe, l,l-cyclo­(leucyl-prolyl), dextrometorfano, Leu-Phe, and d-glucósil-dihidrosfingosina, indicative of peptide release and lipid cleavage, while DG­(17:0/19:0/0:0) [iso2] and parachlorophenol fell abruptly, compatible with the consumption or reimportation of structural diglycerides and detoxification of xenobiotics.

**Figure 7:**
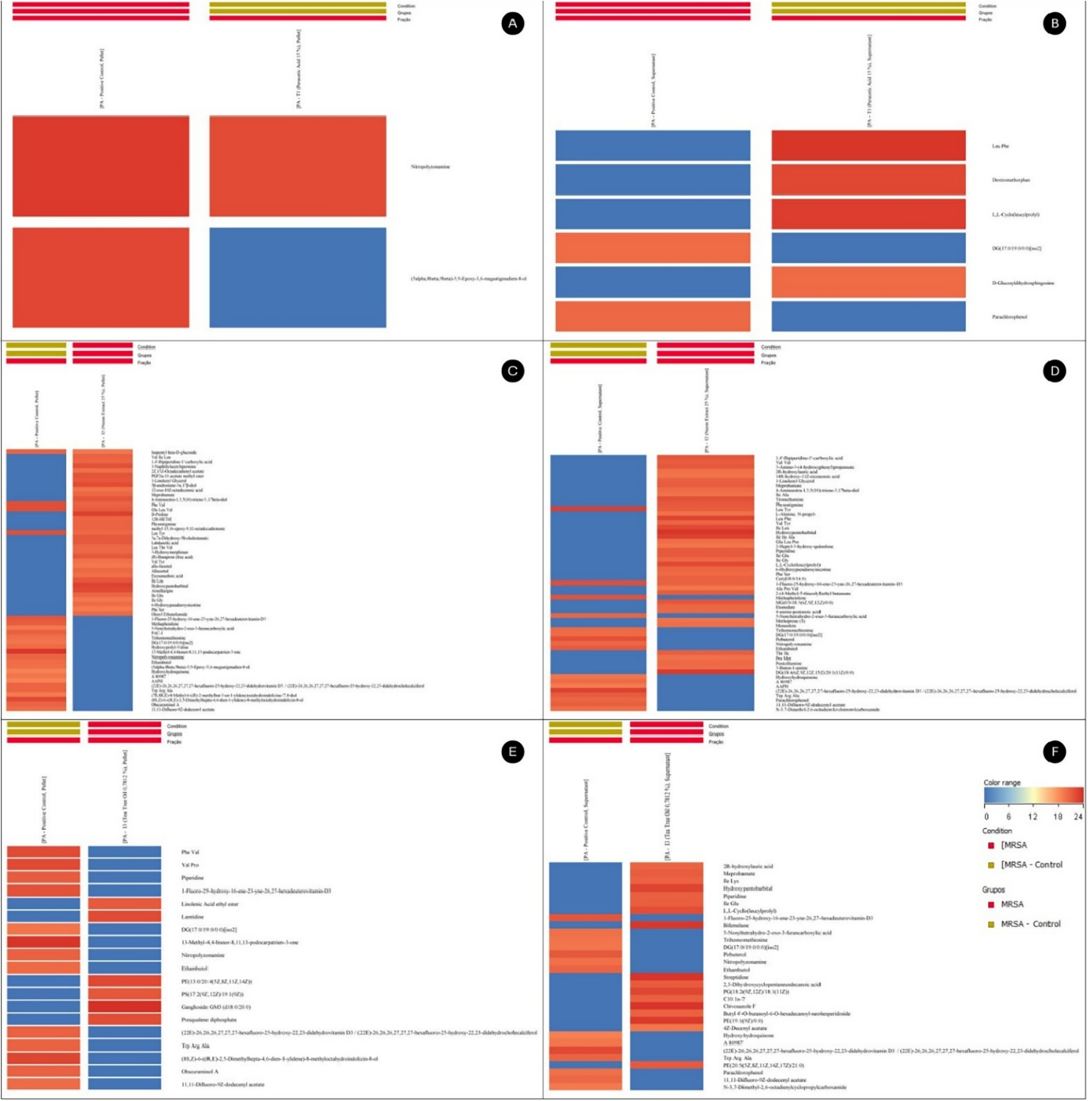
Heatmap representing the relative abundance mean of selected metabolites in pellets and supernatants of PA in T1 (A, B), in I2 (C, D), and in I3 (E, F), respectively.

**Figure 8:**
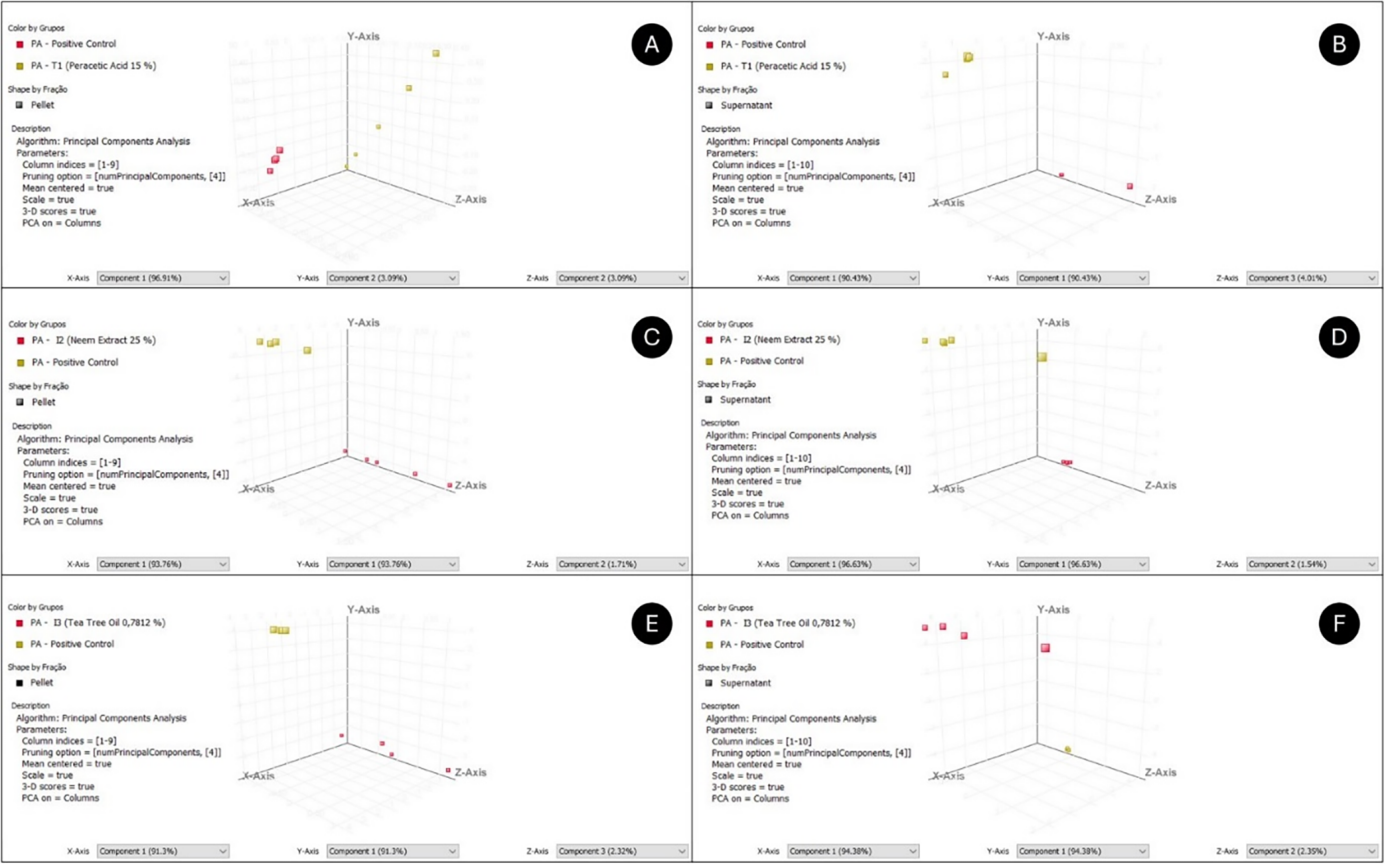
Principal Component Analysis (PCA) score plot based on metabolomic profiles of pellets and supernatants of PA in T1 (A, B), in I2 (C, D), and in I3 (E, F), respectively.

In general, T1 induced a profound oxidative effect in MRSA, altering the respiratory tract, disrupting the membrane, and releasing peroxidation products, while in PA the lipopolysaccharide barrier limits the internal attack, directing the damage to the surface, with extravasation of minor metabolites and partial preservation of the intracellular pool. This fundamental difference shows that the same oxidant acts more lethally on Gram-positive but is still an important disinfection agent against Gram-negative.

Exposure of MRSA to 25% I2 for 1 min initially resulted in 67 intracellular compounds present in all replicates, of which 47 were differentially expressed and 28 bound to central pathways of *S. aureus* (Figures 5c and 6c). A marked reduction in signaling and breathing markers was observed, trans, *trans*-farnesol, Stigmatellin Y, and Tromethamine, and peptides Val-Val, Ile-Lys, and Ala-Pro-Val, as well as free amino acids such as d-proline and physostigmine, suggesting accelerated consumption or selective extravasation. On the other hand, unsaturated lipids and their esters showed a significant increase and included 11-deoxy-PGF_1_α, 1-linoleoyl-glicerol, 2*Z*,13*Z*-octadecadienyl-acetato, oleoyl-ethanolamide, ethyl-linolenic acid, and isoprenoide glycosylated (*S*)-nerolidol-ramnosídeo. The elevation of anionic phospholipids, eicosanodioic acid, and 14,15-EE-8­(*Z*)-E, and nonpolar peptides, Pro-Val-Ile and Ile-Leu, completes the intracellular accumulation profile. In the extracellular medium of MRSA (Figures 5d and 6d), of the 26 common metabolites in all samples, 11 were differentially expressed and associated with bacterial pathways. Eight compounds exhibited increases: the peptides Tyr-Pro-Ala, Arg-Leu-Val, and hydroxyprolyl-valine, the monoglyceride monoolein, the diglyceride DG­(18:1/18:1/0:0), the prostaglandin 17-phenyl-trinor-PGF_2_α-d_4_, the basic amino acid d-ornithine, and the xenobiotic bronchodilator pirbuterol. This accumulation indicates cytosolic extravasation and the detachment of fragments from the bilayer. On the other hand, three metabolites were drastically reduced: the fatty acid oxidation products (2*R*-hydroxylauric acid, octadecyl fumarate) and the β-oxidation intermediate 2,3-dihydroxycyclopentaneundecanoic acid, suggesting their capture or accelerated intracellular degradation for energy generation or recycling of antioxidant cofactors.

In PA, I2 maintained 70 intracellular metabolites (Figures 7c and 8c), 55 were differentially expressed, and 36 were linked to metabolic pathways of lipid biosynthesis, detoxification, and peptide transport. Among those that increased, Val-Ile-Leu, Glu-Leu-Val and d-proline peptides stand out, as well as lipids such as 1-linoleoyl glycerol, PGF_2_α-11-acetate, 12*R*-HETrE, labdanolic acid, and eicosanodioic. On the other hand, compounds linked to membrane integrity and redox metabolism, which include DG­(17:0/19:0/0:0), hydroxyhydroquinone, ethambutol, (5α,8β,9β)-epoxy-megastigmadienol, were reduced, indicating oxidative cleavage of key molecules. In the extracellular medium of PA (Figures 7d and 8d), 75 metabolites were frequent, 55 varied, and 38 were related to bacterial pathways. Large elevations of peptides (Val-Val, Ile-Leu, and Leu-Phe), lipids (monoolein, polyunsaturated DG), and quorum sensing signalers (2-heptyl-3-hydroxy-quinolone), as well as xenobiotics (Meprobamate, Physostigmine) were observed. Parachlorophenol Drop Supports Rapid Detoxification Mechanisms.

In summary, I2 operates by hybrid mechanisms in both pathogens, combining membrane permeabilization, evidenced by peptide extravasation and lipid cleavage, with redox enzymatic inhibition, manifested in the decrease in respiratory cofactors. Although MRSA and PA share changes in bilayer and signaling, MRSA has a response more focused on sphingolipid modulation, and PA exhibits extensive adaptation via quorum-sensing signaling, reflecting the distinct architecture of its cell walls.

Exposure of MRSA to I3 0.78% for 1 min revealed marked changes in both intra- and extracellular media. Of the 59 compounds present in all pellet replicates (Figures 5e and 6e), 15 were differentially expressed, and 6 were part of pathways associated with lipid biosynthesis, signaling, and respiration. Marked reductions in Val-Ile-Leu, phytosphingosine, 1-fluoro-25-hydroxy-vitamin D_3_, and endogenous antibiotic A 80987 were noted, indicating cytoplasmic leakage and cleavage of sphingoidal bases. There was an increase in d-phenylalanine, cardiolipin CL­(20:1/18:2/18:2/18:1), presqualene-diphosphate, fatty acid C10:1 *n* – 7, and ganglioside GM3, which are consistent with signs of attempted membrane reinforcement and reorganization of lipid domains. Also in the pellet, the accumulation of external terpene compounds (lamtidine, eudesmin, patchoula-diene, quinkenoside F1, butyl-neohesperidoside, netilmicin) reinforced the high permeability induced by I3. In the MRSA supernatant (Figures 5f and 6f), 36 metabolites were constant, 7 were significantly altered, and 4 corresponded to bacterial pathways. Only octadecyl fumarate decreased, suggesting rapid reabsorption, while six metabolites increased: the dipeptides Tyr-Leu and Ile-Arg, the phospholipids cardiolipin and PS­(14:0/12:0), the glycosylated terpenoid butyl-neohesperidoside, and a ceramide-derived glycosphingolilipid. This profile confirms terpinen-4-ol-mediated microporation, with selective extravasation of lipophilic and low-weight molecules.

In PA, the intracellular response showed 45 frequent metabolites (Figures 7e and 8e), 19 were differential, and 11 corresponded to critical pathways. Reductions in Phe-Val, piperidine, DG­(17:0/19:0/0:0)­[iso2], nitropolyzonamine, and ethambutol were highlighted, suggesting oxidative cleavage of structural lipids and redox intakes. The elevation of Ganglioside GM3, presqualene-diphosphate, PE­(13:0/20:4), and PS­(17:2/19:1) indicated attempts to repair the bilayer. In the PA supernatant (Figures 7f and 8f), 51 metabolites were filtered, 31 ranged, and 17 were from bacterial pathways. An increase in streptidine, dipeptides (Leu-Phe and Val-Tyr), polyunsaturated phospholipids, and xenobiotics (Meprobamate) was observed, while A 80987 and parachlorophenol decreased, reflecting the export of defense and detoxification compounds.

The profiles converge in the disorganization of the bilayer and in the extravasation of peptides but diverge in extension. While MRSA undergoes rapid lipid remodeling, PA retains part of the intracellular pool. I3 targets both pathogens via micropore formation and the inhibition of respiratory pathways, although the architecture of LPS in PA moderates internal damage.

## 4 Discussion

This study addresses a significant gap in the field of cleaning and disinfection by covering both traditionally marketed products and innovative agents applied to environmental surfaces. Weber et al.^15^ emphasized the importance of a careful evaluation of sanitizing agents, considering not only the appropriate concentration but also the duration of exposure. These parameters are crucial to ensure that the agents achieve the required level of disinfection, helping to control pathogenic microorganisms in various environments.^16,17^

The preliminary analysis showed that traditional active ingredients demonstrated efficacy at lower concentrations and shorter exposure times, which differed from the innovative products, such as I1 and I2, which required 100 and 25% concentrations, respectively. Previous studies did not explore the antibacterial activity of pine oil in the tested strains at high concentrations.

Neem extract, according to a study by Ali et al. (2021), demonstrated potential antibacterial activity at concentrations between 25, 50, and 100%. Similarly, the present study showed that the PA strain exhibited resistance to 7 out of the 10 active ingredients tested. The resistance to quaternary ammonium, commonly used in hospitals, was significant due to growth at high concentrations for up to 10 min. Beier et al. (2015) suggested that this resistance might be linked to efflux mechanisms, allowing *P. aeruginosa* (PA) to resist quaternary ammonium, even under challenging conditions.

For other agents, PA’s resistance to biguanide and innovative agents may be related to specific mechanisms, including porin-dependent inhibition, development of an impermeable outer membrane, and strain-dependent responses. Despite these resistance mechanisms, the efficiency test demonstrated that 9 out of 10 active ingredients effectively controlled PA, likely due to the higher concentrations used in the macrodilution method, which is more robust than the MBC test. According to Rosa et al.,^18^ using a microdilution test (MIC/MBC) prior to a macrodilution test helps ensure consistency and reliability in results.

Neem (*Azadirachta indica*) is known for its insecticidal properties, but it also has antimicrobial activity due to bioactive compounds such as nimbidine and nimbolide.^19–22^ Tea tree oil (I3) also showed promising results, reducing more than 99.99% of bacteria at the lowest concentration tested (0.7812%). Its antimicrobial action is due to terpinen-4-ol, a component that is effective in disrupting bacterial membranes. However, tea tree oil showed inefficacy against MRSA and SA strains, which aligns with findings by May et al. (2000), who noted the difficulty in inactivating *S. aureus* at similar concentrations.

Orange oil (I4), at 1%, exhibited greater effectiveness against Gram-negative bacteria, possibly due to its phenolic components, especially limonene. This aligns with findings by Velázquez-Nuez et al. (2013) and Souza (2022), who noted that orange oil’s selectivity results in more effective inhibition of Gram-negative bacterial growth. Pine oil (I1), however, showed no significant efficiency in any of the strains tested, even at 0.4%, consistent with Nisca et al. (2021), who found low effects of pine oil on Gram-negative bacteria.

Lactic acid (I5) was effective in controlling certain strains but showed a limited efficacy against AB bacteria. Lactic acid’s antimicrobial action occurs as it crosses microbial cell membranes, lowering intracellular pH and leading to bacterial death. However, the persistence of AB bacteria in the presence of I5, I1, and I4 may be attributed to resistance mechanisms such as biofilm formation, genetic mutations, and plasmid-mediated resistance.^24,25^

Several innovative agents (I2, I3, I4, and I5) were effective against critical strains (KPC, CJ, and ST) of public health concern. The presence of compounds such as nimbidine, terpinen-4-ol, and d-limonene, which interfere with essential bacterial processes, likely contributed to these agents’ efficacy.^23,26^ The *in situ* analysis highlighted the practical effectiveness of certain agents under real-world conditions, especially given the resistance of biofilms, which are notoriously harder to penetrate with antimicrobial agents.^27,28^

The *in situ* test demonstrated the effectiveness of products T1, T4, I2, I3, and I4, with a bacterial reduction of over 99.99%. However, the inefficiency of quaternary ammonium compounds (T2 and T3) was significant. Despite their recognized antimicrobial efficacy, resistance mechanisms like those seen in antibiotic resistance might have compromised their performance.^29^ The concentration used in this study (0.8%) was lower than that in previous studies, possibly contributing to the lack of efficacy. Colen et al. (2020) emphasized the complexity of determining optimal concentrations to ensure disinfection effectiveness.

Chlorhexidine digluconate (T5) at 0.15% was also ineffective in the *in situ* test, echoing results from a University of São Paulo study that found chlorhexidine digluconate (0.12%) effective at biofilm removal but falling short of ANVISA’s 99.99% disinfection requirement.^30,31^ Chlorhexidine’s antimicrobial action is driven by electrostatic interactions between its cationic molecules and the negative charge of bacterial cell walls, but this mechanism may be compromised by biofilms, which create barriers that limit the effectiveness of such agents.^32,33^

Peracetic acid (T1) and biguanide (T4) showed successful disinfection at concentrations of 0.1 and 0.4%, respectively. Peracetic acid acts by oxidizing sulfhydryl and sulfur bonds in microbial cell walls, while biguanide disrupts biofilm formation and bacterial growth. These attributes make them effective biocides, especially against multidrug-resistant strains.^34^ However, the innovative ingredients I1 (pine oil) and I5 (lactic acid) were less promising, possibly due to the specific application conditions and concentrations used in this study.^35^

Pine oil’s antibacterial activity is largely attributed to high concentrations of α- and β-pinene.^36^ However, in this study, pure pine oil showed no effect. The antimicrobial efficacy of pine oil can vary, depending on the pine species and the concentrations of its compounds. Carpenter et al.^37^ demonstrated that higher concentrations of lactic acid (1–2%) effectively reduced bacterial populations on carcasses, but in this study, the lower concentration of 0.4% may explain the lack of efficacy.

The presence of biofilms on the tested surfaces may also have hindered the effectiveness of these agents. Biofilms can create protective barriers that limit the penetration of antimicrobial agents, requiring higher concentrations or alternative methods to disrupt them effectively.^38,39^

The study of the mechanisms of action was focused on the most efficient compounds that corresponded to T1, I2, and I3 in two bacteria representing the group of Gram-positive (MRSA) and Gram-negative (PA). In general, it was observed that each agent promoted distinct responses in both species, both in magnitude and in the mechanisms involved.

T1 showed rapid oxidizing action, characterized by the induction of lipid peroxidation and disorganization of the phospholipid bilayer in both species. In MRSA, there was intracellular accumulation of saturated ceramides, such as Cer­(d18:0/14:0), and peptides such as Ala-Leu-Ala, suggesting an attempt to reinforce the membrane as an adaptive response to oxidative lesions, a phenomenon already described in bacteria exposed to potent sanitizers.^40^ The emergence of compounds such as Stigmatellin Y, a byproduct of ubiquinone, reinforces the hypothesis of direct interference in electronic transport, culminating in energy collapse.^41^ Extracellularly, the data reinforce this dynamic. In MRSA, the abrupt release of intracellular peptides and oxidized lipids ((+)-3-hydroxy behenic) was observed, showing loss of membrane selectivity. In PA, the effects were similar, with release of l,l-cyclo­(leucilprolyl) and dextromethorphan in the supernatant, indicating disruption of the lipid bilayer and failure of the transport systems. The reduction of intracellular nitropolyzonamine confirms the attack on antioxidant pathways, suggesting exhaustion of the BP defensive response, as already described by Elghali et al. (2024) in response to reactive oxygen species. The T1 mechanism is therefore markedly oxidative in both species, with membrane initiation and progression to bioenergetic breakdown. However, in PA, which has a double membrane, permeabilization seems to be less efficient, reflected in the lower amount of differentially expressed metabolites.

I2, rich in terpene compounds, demonstrated broader action on the MRSA and PA metabolic pathways. In MRSA, there was intracellular accumulation of unsaturated lipids and a decrease of essential peptides, showing β-oxidation blockade and protein degradation, respectively. The fall of Stigmatellin Y confirms inhibition of the bc_1_ complex, affecting electronic transport.^41,42^ The extracellular profile revealed release of peptides such as Tyr-Pro-Ala and hydroxyprolyl-valine, as well as glycerides such as monoolein, corroborating the hypothesis of cell wall disruption by lipid permeabilization. In PA, I2 induced accumulation of peptides and lipids in the supernatant and depletion of intracellular reserves such as ethylbutol and nitropolyzonamine. The Val-Ile-Leu↑/DG­(17:0/19:0)↓ pattern stood out as a robust marker of the action. This profile is compatible with the literature on the cytotoxic effects of terpenes on bacteria, which include membrane insertion, increased fluidity, and disorganization of functional domains.^43–45^ Unlike T1, I2 induces a more gradual collapse, associated with permeabilization and metabolic disturbances that go beyond the membrane, reaching signaling, detoxification, and lipid regeneration pathways, being more intense in PA. The strong release of Tromethamine and Physostigmine suggests advanced redox stress in PA, with repair failure and intracellular regulation.

I3 produced biphasic changes in both species. In MRSA, the effect began with the loss of peptides such as Val-Ile-Leu and reduction of phytosphingosine, followed by an attempt to reconstruct the membrane with an increase in cardiolipin and gangliosides. The insertion of terpenes into the lipid bilayer of MRSA can form transient pores, promoting selective extravasation, compatible with proposed action models for essential oils.^46^ The release of anionic lipids in the supernatant, such as phosphatidylserine, indicates the loss of membrane asymmetry, a typical event of irreversible damage. In PA, the pattern was similar, with intracellular reduction of peptides and diglycerides, followed by elevation of gangliosides and prenylation as presqualene diphosphate, indicative of compensatory pathways. The release of compounds such as Ile-Lys and l,l-cyclo­(leucilprolil) in the medium and the drop in DG­(17:0/19:0) point to a progressive collapse of the barrier and energy reserves. The action of I3 is consistent with studies that demonstrate that tea tree terpenes promote increased permeability and disorganization of bacterial liposomes and micelles.^40^ Despite the similarity in the routes hit by I3 in both species, the adaptive response of PA seems more robust, as evidenced by the increase in complex structural compounds. Still, the magnitude of the loss of bioactive metabolites suggests that such a response is not sufficient to counteract the bactericidal effect.

In our study, we observed that despite the bactericidal effect identified in the MBC test (Table 3), the efficiency test demonstrated maintenance of the bacterial count in MRSA and an increase in AS (Figure 3b). This occurred because, even with the same exposure time, the tests evaluate different phenomena. The MBC determines the lowest concentration capable of reducing the bacterial population, accounting for idealized postexposure growth (rich medium, without obstacles to the agent). The efficiency test, on the other hand, measures the immediate reduction of colonies after 1 min, reflecting the behavior of the product under more real conditions of the test, and can reveal populations that, although damaged, remain viable soon after exposure,^47^ complementing the response identified in MBC. The data obtained in the metabolomic analysis of MRSA under the effect of I3 provide a more robust response to the divergences identified in the MBC and in the efficiency test. Despite the indications of microporation and high permeability induced by I3, there is also a membrane stabilization response, demonstrating that although there is significant damage, not the entire population quickly deviates, probably due to a difference in the expression of the membrane stabilization response. This reinforces that the MBC detects potential bactericidal action but the efficiency test exposes the real loss of immediate population viability.

The three sanitizing agents tested presented distinct and complementary mechanisms. T1 acts in a direct oxidative manner, promoting lipid peroxidation and the release of intracellular metabolites. I2 combines terpene action with induction of bioenergetic breakdown and depletion of repair routes, while I3 acts mainly by physical disorganization of the membrane, initiating porosity and exfoliation of lipid domains. In terms of species, PA, due to its double membrane and greater lipid complexity, exhibits a broader adaptive response but also is more vulnerable to compounds that interfere with multiple pathways, such as I2. MRSA, on the other hand, succumbs more quickly to mechanisms that promote the direct oxidation of the bilayer, as observed with T1.

In conclusion, this study underscores the need for adaptable and specific cleaning and disinfection strategies to ensure effectiveness. The results highlight the complexity of achieving the desired efficacy, particularly in meeting regulatory standards such as those set by ANVISA. The innovative products, while promising, may require further refinement, particularly in their application in hospital and industrial settings. Additionally, the study emphasizes the importance of adjusting biocide concentrations to optimize disinfection *in situ*, as variations in concentration can significantly impact effectiveness.

## 5 Conclusion

Our study proved the importance of preliminary analysis (MBC) combined with a more representative test at the sampling level (efficiency test) in detecting the best concentration to be applied in cleaning processes, considering the specific condition of each environment. The association with the *in situ* test showed that the most promising compound for bacterial control was peracetic acid, due to the lower concentration (0.1%) used and the proven effect in the removal of bacterial load at levels not detectable in 1 min. As innovative alternatives, we identified tea tree and neem, which at 0.7812 and 25%, promoted the desired efficiency. In metabolomics, the three compounds demonstrated rapid and differential action on Gram-positive and Gram-negative bacteria, affecting essential metabolic pathways and highlighting promising targets for bacterial control.

## Supplementary Material



## Data Availability

All data supporting the findings of this study are available in the article itself (text, tables, and figures) and in the Supporting Information.

## Notes

Minimum bactericidal concentration (MBC), exposure times, and resistance profiles of bacterial strains against the chemical agents tested (Table S1) (XLSX)

## Abbreviations

ANOVA: analysis of variance
Anvisa: Brazilian Health Regulatory Agency
ATCC: American Type Culture Collection
CCDA: Ágar Campylobacter Blood-Free Selective Medium
CFU: colony-forming unit
CH: Clinical Hospital
VH: Veterinary Hospital
PI: poultry industry
MBC: minimum bactericidal concentration
MDR: multidrug-resistant
PCA: plate count agar
